# Efficacy and safety of anlotinib in patients with desmoid fibromatosis: a retrospective analysis

**DOI:** 10.3389/fonc.2024.1399574

**Published:** 2024-05-14

**Authors:** Mengzhang Xie, Qi Huang, Taojun Gong, Yitian Wang, Zhuangzhuang Li, Minxun Lu, Yi Luo, Li Min, Yong Zhou, Chongqi Tu

**Affiliations:** ^1^ Department of Orthopedic Surgery and Orthopedic Research Institute, West China Hospital, Sichuan University, Chengdu, China; ^2^ Model Worker and Craftsman Talent Innovation Workshop of Sichuan Province, Chengdu, Sichuan, China; ^3^ Operating Room, West china Hospital, Sichuan University/West China School of Nursing, Sichuan University, Chengdu, Sichuan, China

**Keywords:** desmoid fibromatosis, anlotinib, tyrosine kinase inhibitor, targeted therapy, dosage adjustments

## Abstract

**Introduction:**

Desmoid fibromatosis is an aggressive fibroblastic neoplasm with a high propensity for local recurrence. Targeted therapy for Desmoid fibromatosis represents a novel avenue in systemic treatment. Anlotinib, a novel multitargeted angiogenesis inhibitor, represents a novel approach for targeted therapy. Therefore, this study aims to assess the efficacy and safety of anlotinib in patients with Desmoid fibromatosis.

**Methods:**

We retrospectively gathered the clinical medical records of Desmoid fibromatosis patients who underwent anlotinib treatment between June 2019 and November 2023 at our center. Anlotinib was initiated at a daily dose of 12 mg and adjusted based on drug-related toxicity. Tumor response was evaluated using the Response Evaluation Criteria in Solid Tumors 1.1 criteria. Progression-free survival served as the primary endpoint and was analyzed utilizing the Kaplan–Meier method.

**Results:**

In total, sixty-six consecutive patients were enrolled. No patients achieved a complete response; however, fourteen patients (21.21%) exhibited a partial response, while forty-six patients (70%) experienced disease stability. Progressive disease was observed in 6 patients (9.10%), and the progression-free survival rates at 12 and 36months were 89.71% and 82.81%, respectively. The disease control rate was 90.91%, while the objective response rate was 21.21%.

**Conclusion:**

Anlotinib proves effective in managing recurrent and symptomatic patients with Desmoid fibromatosis. However, the toxicity profile of anlotinib presents a higher risk of Hand-Foot Skin Reaction and hypertension. Therefore, given that 41.67% of patients were subjected to dose adjustments associated with the initial dose of 12 mg, implementing dosage reductions may help balance efficacy with side effects.

## Introduction

Desmoid fibromatosis (DF), also referred to as aggressive fibromatosis, deep fibromatosis, or desmoid-type fibromatosis, is a type of soft tissue mesenchymal neoplasm (1). DF is a rare disease constituting <3% of soft tissue neoplasms with a high incidence age of 30 to 40 years and can be found at virtually any anatomical location (2–4). DF represents a clonal fibroblastic proliferation that emerges in deep soft tissues and is distinguished by infiltrative growth and a propensity for local recurrence but an inability to metastasize (5). In studies of DF patients, the 5-year local recurrence survival rate was 47.1% among 88 patients after surgery (6). Additionally, spontaneous regression was noted in 27 out of 108 patients (25%) (7). Therefore, DF is highly variable, and its prognosis is often unpredictable, but tumor size, location, growth rate and invasion of surrounding tissues are related to its prognosis (8, 9).

For the management of DF, the concepts of diagnosis and treatment emphasize individualization and multidisciplinary management. For asymptomatic DF in the extremities, flexible active surveillance (AS) is the preferred treatment approach (7, 10). For tumors that cause symptoms, impair function, or pose a threat during AS, systemic therapy and surgical treatment are often considered.

Current guidelines recommend tyrosine kinase inhibitors (TKIs) as the drug of choice for systemic treatment in DF patients who have unresectable, progressive, or recurrent tumors (1). In contrast to the toxic effects of cytotoxic chemotherapy, TKIs are usually continued until intolerance or disease progression occurs. Additionally, TKIs have positive clinical results, and the use of sorafenib has entered the phase 3 clinical trial stage (11–14).

Although surgery is not considered a first-line option due to the high local recurrence rate and the presence of a surgical site and R1 surgical margin negatively impacts patient prognosis postsurgery, surgery remains the viable choice for rapidly progressing tumors located in important anatomical structures (1, 6, 15–17). Current evidence supports the notion that combining RI/R2 resection margin surgery with radiotherapy in patients with extra-abdominal DF may effectively reduce postoperative recurrence (18); however, it is important to consider the drawbacks of adjuvant radiotherapy, such as prolonged treatment duration and potential scarring after radiation therapy (19, 20). The use of preoperative medication does not appear to significantly reduce the rate of postoperative recurrence (6). However, there is limited research on whether TKIs can be employed as adjunctive therapies in surgical treatment to effectively decrease the occurrence of postoperative recurrence.

Anlotinib is a novel oral multitargeted receptor tyrosine kinase inhibitor independently developed in China that targets vascular endothelial growth factor receptor (VEGFR), fibroblast growth factor receptor (FGFR), platelet-derived growth factor receptor (PDGFR), and c-Ki (21). This drug was approved by China for marketing in 2018. For unresectable or metastatic soft tissue sarcoma, anlotinib treatment has promising efficacy and manageable toxicity (22–24). In addition, anlotinib can also be used as an adjuvant therapy to reduce the postoperative recurrence rate of hepatocellular carcinoma and gastric adenocarcinoma (25, 26). However, very few studies on DF have been conducted.

Therefore, based on our previous study of anlotinib in DF patients, anlotinib can slow the progression of DF with acceptable safety (27). However, the effects of anlotinib on other treatment methods, dosages and long-term effects on patients still need to be further studied. Subsequently, we extended the use of anlotinib in patients with DF. In this study, we conducted a retrospective review of DF patients treated with anlotinib at our institution, aiming to evaluate both its safety and efficacy profiles. Additionally, we investigated the recurrence rate following surgery among patients receiving combined surgical treatment with anlotinib.

## Patients and methods

### Patient selection

We retrospectively analyzed desmoid fibromatosis patients treated with anlotinib in the Department of Orthopedics at our hospital between June 2019 and November 2023.

The following is a list of inclusion criteria for patients: (1) confirmation of tumors through pathological examination; (2) manifestation of symptomatic and progressive tumors; (3) recurrence of tumors or the presence of an unresectable primary tumor; and (4) voluntary refusal by patients to undergo radiotherapy, chemotherapy, or surgery. (5) Having at least one measurable disease as defined by the Response Evaluation Criteria for Solid Tumors (RECIST) 1.1. (6) No systemic antitumour treatments were administered within 4 weeks before enrollment.

Tumor assessments were performed using magnetic resonance imaging (MRI) within two cycles or more frequently based on the specific development of the tumor. The term “unresectable disease” is defined as the condition in which resection of the tumor results in an unacceptable morbid outcome. Data on patient and tumor characteristics, including age, sex, presentation status (primary or recurrent), tumor size, tumor site, prior therapeutic history, initiation date of anlotinib treatment, rationale for discontinuation, treatment dosage and associated adverse reactions, date of progression, and date of death, were collected.

### Treatment and evaluation

Patients received anlotinib at an initial dose of 12 mg once daily; in a 2-week on/1-week off treatment cycle that lasted three weeks. However, dose reduction (to 10/8 mg) was allowed if the patient showed a history of intolerable or uncontrolled drug-related toxicity based on considerations of toxicity and tumor response according to Response Evaluation Criteria in Solid Tumors (RECIST) 1.1. Treatment was temporarily stopped when drug-related side effects could not be managed through dose reduction and symptomatic treatment. Once the dose was reduced, it could no longer be increased. Before treatment initiation, it is necessary to establish a baseline, which includes both a physical examination and imaging evaluation. Follow-up visits primarily occurred at an outpatient clinic.

In our study, progressive disease (PD) was determined through MRI conducted every three months. The primary endpoint, progression-free survival (PFS), was defined as the duration from the commencement of treatment to tumor progression, death, or the last follow-up. The objective response rate (ORR) was determined as the ratio of patients who achieved a complete response (CR) to those who achieved a partial response (PR). The disease control rate (DCR) was identified as the percentage of patients showing no recorded tumor progression. Drug-related adverse effects were categorized and graded based on the National Cancer Institute Common Terminology Criteria for Adverse Events (version 5.0).

In the actual application process, dosage determination requires comprehensive consideration of factors such as patient age, weight, severity of illness, imaging findings, and symptoms. The initial assessment should evaluate whether the size and growth rate of the tumor are acceptable. If there are no special circumstances, the treatment should begin with a dosage of 12 mg. Dosage adjustments require comprehensive consideration of drug-related toxicity and tumor response, assessed according to the National Cancer Institute Common Terminology Criteria for Adverse Events (version 5.0) and the RECIST 1.1. In the case of stable SD or PR, for Grade 1–2 adverse reactions, observation or symptomatic treatment with medication should be initiated for 4 cycles. If the adverse reactions are manageable and the patient tolerates them subjectively, treatment with the current dosage can be continued. If inadequate control of adverse reactions occurs during treatment, dosage adjustment or discontinuation should be considered. In principle, if the tumor does not progress and the patient tolerates drug-related adverse reactions, treatment with the current dosage may continue. However, dosage reduction should be primarily based on the patient’s request. For Grade 3 or higher adverse reactions, symptomatic treatment should be administered for 2–3 cycles, or the dosage should be directly reduced. In the case of adverse reactions such as bleeding or cardiac issues, dosage reduction or even discontinuation may be necessary.

For hypertension, early treatment with ACE inhibitors (ACEI) or angiotensin II receptor blockers (ARBs) is recommended. If ineffective, combination therapy with other antihypertensive agents such as calcium channel blockers, diuretics, and beta blockers should be considered. If blood pressure normalizes, reduction or discontinuation of relevant antihypertensive medications is warranted. Management of hand-foot syndrome primarily focuses on skin protection. This includes wearing thick insoles, gloves, or using moisturizers and corticosteroid creams for local care. If these measures are ineffective, dosage adjustment should be considered. For diarrhea, dietary changes and increased fluid intake are recommended. If diarrhea persists, antidiarrheal medications should be used. In cases of severe diarrhea, dose reduction or discontinuation of medications may be necessary.

This study was performed according to the principles embodied in the Declaration of Helsinki and the Institutional Review Board of Sichuan University West China Hospital. Written informed consent was obtained from all patients prior to their enrollment in the study.

### Statistical analysis

For descriptive data, categorical variables were converted to frequencies (percentages), while continuous variables were converted to medians and interquartile ranges (IQRs). Survival analysis, including estimation of PFS, was conducted using the Kaplan-Meier method to generate survival curves. The Clopper–Pearson method was used to calculate the 95% confidence intervals for the overall objective ORR and DCR. Statistical significance was set at p < 0.05, and statistical analysis was performed using GraphPad Prism version 9.0 (GraphPad Software, La Jolla, CA) and SPSS version 27.0 (IBM Corporation, Armonk, NY, USA).

## Results

### Patient characteristics

As detailed in **Table 1**. From June 2019 to November 2023, 66 patients (25 men and 41 women) with DF were included, with a median age of 33 (13–77) years. Pathologic diagnoses were confirmed by the pathology department of our hospital. The most common anatomical locations of DF were the gluteal region (13.6%), upper arm (12.1%), thigh (9.09%), and various other areas. Previous therapies were diverse, with the exception of 28 patients (37.9%) who had not received any therapy before beginning treatment. Locally recurrent tumors were common, with 43.9% experiencing recurrence after surgery, 21.21% after multiple surgeries, and a few deemed inoperable or declined surgery. Before receiving anlotinib treatment, 13.64% of patients underwent Apatinib therapy.

**Table 1:** Baseline characteristics of the patients with anlotinib.

Parameter	Patients (%)
Age	
Median	33
Range	13-77
Gender	
Female	41
Male	25
Site of tumor	
Limbs and girdle	51
Abdominal wall + mesentery	4
Others*	11
Number of previous surgeries	
0	31
1	28
2 or more	7
Previous medical treatment	
Chemotherapy	0
Apatinib therapy	9

*Others, chest wall, lumbar region, axillary region, and neck.

### Efficacy of anlotinib

**Figure 1:**
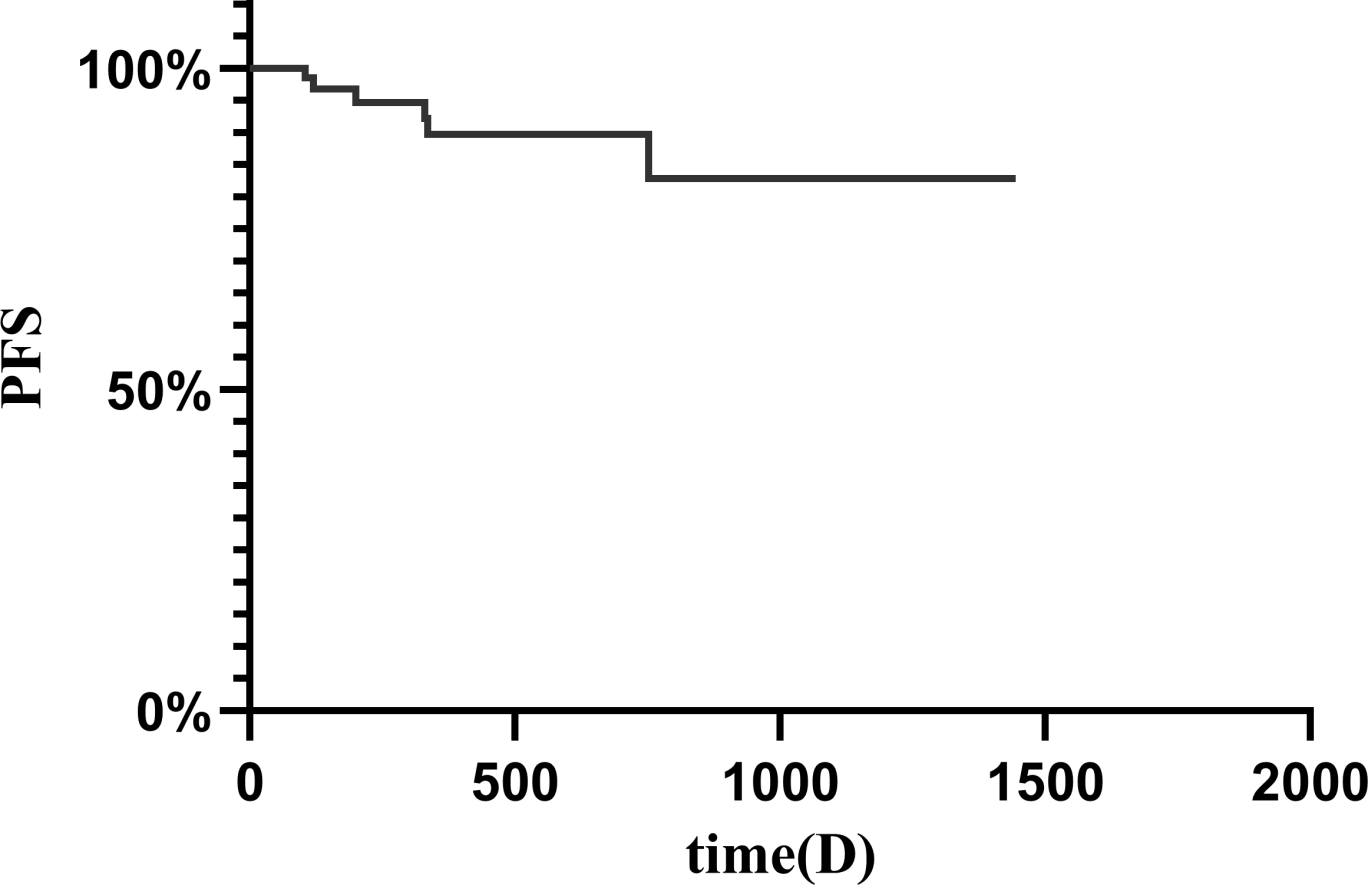
Kaplan-Meier curve demonstrating progression-free survival in all patients.

**Figure 2:**
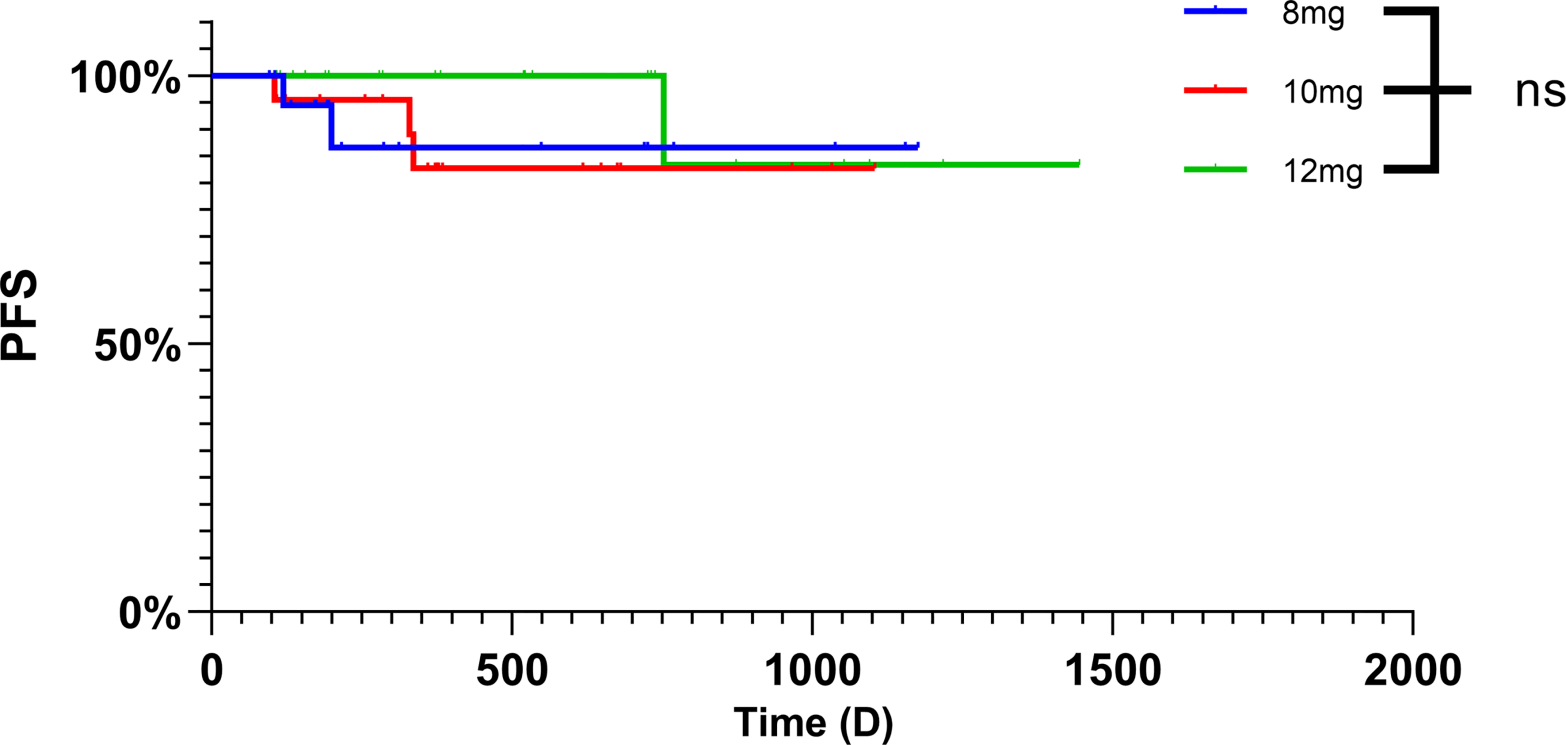
Kaplan-Meier curve demonstrating progression-free survival in three initial dose.

The clinical characteristics of patients treated with anlotinib are presented in **Table 2** and **Figure 1**. The response rates for different initial doses of anlotinib are outlined in **Table 3** and **Figure 2**. The median follow-up period was 373 (96–1445) days, and no patient succumbed to the disease.

**Table 2:** Response rates of anlotinib.

Parameter	N (%)
Number	66
CR	0
PR	14(0.21)
SD	46(0.70)
PD	6(0.10)
ORR	14(0.21)
DCR	60(0.91)

ORR=CR+PR; DCR=CR+PR+SD.

**Table 3:** Response rates of different initial doses of anlotinib.

	12	10	8
Number	24	22	20
CR	–	–	–
PR	8(0.33)	5(0.23)	1(0.05)
SD	15(0.63)	14(0.64)	17(0.85)
PD	1(0.04)	3(0.14)	2(0.10)
ORR	8(0.33)	5(0.23)	1(0.05)
DCR	23(0.96)	19(0.84)	18(0.9)

**Figure 3:**
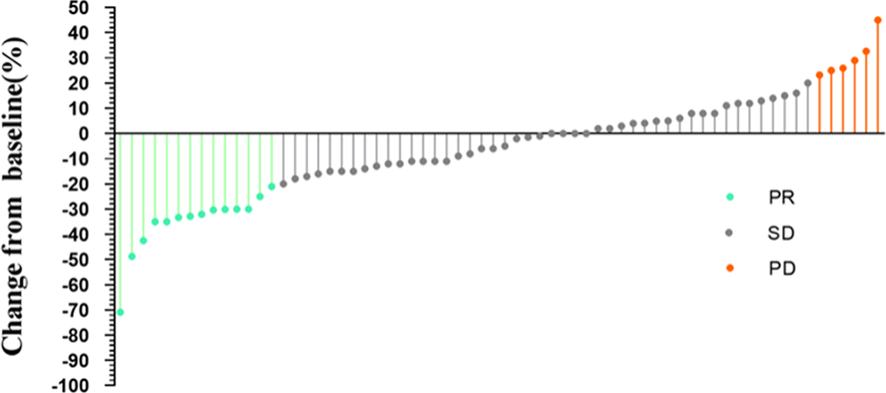
Change from baseline.

The results showed that among the 66 patients, 21.2% achieved a PR, with 46 patients exhibiting SD (**Figure 3**). The median tumor reduction was 15% (Q1: 11.00%; Q3: 31.20%), and no patients achieved a CR. The overall DCR for DF patients was 93.94% (95% confidence interval [CI] 0.83–0.96), and the ORR was 21.21% (95%CI 0.13–0.32).

**Figure 4:**
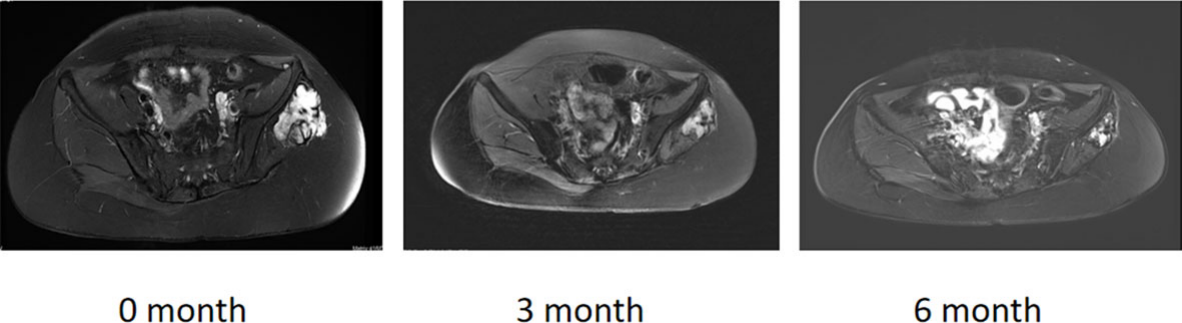
A 28-year-old patient with 6 months of anlotinib.

The PFS rates were 96.80% and 94.69% at 4 and 8 months, respectively, with a 1-year PFS of 89.71% and 3-year PFS of 82.81%, but the median PFS was not reached at the time of analysis. Six patients (9.09%) developed PD; among these six patients, one had three previous recurrences and experienced 23.2% tumor enlargement after 11 months of follow-up while receiving 10 mg of anlotinib. We recommended surgery, but the patient opted to continue oral anlotinib-targeted therapy. During the follow-up, the tumor size decreased and stabilized to SD, so oral anlotinib treatment was continued. Two PD patients received anlotinib immediately after biopsy diagnosis, PD occurred after 3 months of follow-up, and surgical treatment was recommended; however, the patients had not received surgical treatment at the time of analysis. Another previously untreated patient showed disease progression after completing one cycle of 10 mg of anlotinib, and further treatment decisions were pending at the time of analysis. One patient experienced recurrence after hemi-pelvic amputation and opted for imatinib instead of undergoing secondary surgery due to anatomical limitations. The last patient, following recurrence, chose radiotherapy and was subsequently lost to follow-up. Additionally, one patient discontinued four cycles of anlotinib due to other surgeries, experienced disease progression during the drug withdrawal period, resumed the original dose of anlotinib after surgery, achieved stable disease (SD) three months later, and continued oral anlotinib treatment. MRI images of several patients with desmoid tumors in different locations, assessed as PR after different courses of medication, are shown in **Figure 4** and **Supplementary Figures S1**–**3**.

### Tolerability and adverse effects

The drug-related adverse effects of anlotinib are presented in **Table 4**. A total of 48 (72.7%) patients experienced adverse drug reactions. The most common toxicity observed was hand-foot skin reaction (HFSR) (n=17 25.6%), followed by hypertension (n=9 13.63%), diarrhea (n=7 10.6%), and hoarseness (n=5 7.57%). These adverse events were generally mild (grades 1–2) and were effectively managed with symptomatic therapy or dosage adjustments. The grade 3 adverse events included hand-foot sin syndrome (n=2, 3.03%) and hypertension (n=2, 3.03%), which were controlled by the discontinuation of medication.

**Table 4:** The drug-related Adverse effects of anlotinib.

Adverse Event	Total, n (%)	Grade1	Grade2	Grade3-4
Hand foot skin syndrome	17	5	7	2
Hypertension	9	6	1	2
Diarrhea	7	3	4	0
Hoarse	5	5	0	0
Oral ulcers	2	1	1	0
Rash	2	2	0	0
soreness	2	2	0	0
Local pain	1	0	1	0
Epistaxis	1	1	0	0
Pharyngalgia	1	0	1	0
Fatigue	1	0	1	0
No adverse reactions	18	–	–	–

At the time of the analysis, 34 (51.51%) patients were still receiving anlotinib, but 32 (48.48%) patients discontinued it for various reasons. Four patients (6.06%) discontinued anlotinib due to disease progression, 5 (7.58%) patients stopped treatment because of intolerable side effects, two patients recovered to 10 mg without significant side effects during drug withdrawal and continued to receive 10 mg treatment, and twenty-three patients (34.85%) autonomously chose to discontinue medication. Six of them asked for surgical intervention after tumor reduction, followed by active postoperative monitoring. After experiencing recurrence-free intervals while on medication, 13 patients decided to undergo proactive surveillance. Notably, two patients stopped medication after three months, resulting in recurrence one year later. Another 2 patients discontinued medication due to financial constraints, and 1 patient was off medication for 3 months for other surgeries. **Table 5** summarizes anlotinib dose modifications and discontinuations based on the initial dose. In accordance with the recommended dosage, a 12 mg dose was initiated in 24 patients. Twenty-three patients experienced adverse drug reactions, 10 of whom needed to reduce the drug dose, and 4 patients discontinued the drug because of side effects. Among the 22 patients who received an initial dose of 10 mg, 15 experienced adverse drug reactions. Nine patients reduced the drug dose to 8 mg, which was discontinued in only 1 patient (**Table 6**).

**Table 5:** Anlotinib dose modifications.

Parameter	n = 66 (%)
Initial dose of anlotinib(n=66)	
12mg	24
10mg	22
8mg	20
Patients with initial dose 12 mg (n = 24)	
Stopped due to toxicity	4
Stopped due to autonomous choice	5
Stopped due to PD	0
Dose reduction done	10
Continued at initial dose	5
Patients with initial dose 10 mg (n = 22)	
Stopped due to toxicity	1
Stopped due to autonomous choice	9
Stopped due to PD	2
Dose reduction done	3
Continued at initial dose	7
Patients with dose 8 mg (n = 20)	
Stopped due to toxicity	0
Stopped due to autonomous choice	9
Stopped due to PD	2
Dose reduction done	0
Continued at initial dose	9

**Table 6:** Anlotinib dose-associated adverse reactions.

Adverse Event	Total	Grade1	Grade2	Grade3-4
The drug-related Adverse effects of anlotinib in 12 mg				
Hand foot skin syndrome	9	2	5	2
Diarrhea	3	1	2	0
Hypertension	3	0	2	1
Hoarseness	3	3	0	0
Oral ulcers	1	0	1	0
Rash	1	1	0	0
Pharyngalgia	1	0	1	0
soreness	1	1	0	0
Fatigue	1	0	1	0
Epistaxis	0	0	0	0
Local pain	0	0	0	0
No adverse reactions	1	–	–	–
The drug-related Adverse effects of anlotinib in 10 mg				
Hand foot skin syndrome	3	2	1	0
Hypertension	3	1	1	1
Hoarseness	2	2	0	0
Diarrhea	2	1	1	0
Local pain	1	0	1	0
Oral ulcers	1	1	0	0
Epistaxis	1	1	0	0
Rash	1	1	0	0
soreness	1	1	0	0
Pharyngalgia	0	0	0	0
Fatigue	0	0	0	0
No adverse reactions	7	–	–	–
The drug-related Adverse effects of anlotinib in 8 mg				
Hand foot skin syndrome	5	4	1	0
Diarrhea	2	1	1	0
Hypertension	3	3	0	0
No adverse reactions	10	–	–	–

## Discussion

Systemic therapy plays a predominant role in cases of DF manifesting symptoms or progressing after clinical observation, particularly when it is unresectable. Within the domain of systemic therapy, antiangiogenic drugs play a pivotal role. Antiangiogenic therapy has demonstrated promising tolerable toxicity and efficacy in advanced soft tissue sarcomas.

In this study, the effectiveness and tolerability of anlotinib were evaluated in a real-world setting.

In the evaluation of the effectiveness of anlotinib, it is crucial to include references to imatinib, sunitinib, and sorafenib because clinical studies of anlotinib are lacking. Imatinib was the primary drug administered for DF. Additionally, case reports indicated that imatinib exhibited no discernible toxic effects and notably alleviated patient discomfort (28). In cases of imatinib resistance, sunitinib serves as a viable alternative.

Our results indicate that the PFS rates were 96.80% and 94.69% at 4 and 8 months, respectively, with a 1-year PFS of 89.71%. However, in a prospective phase II trial, fifty-one patients were treated with imatinib. PFS rates were 94% and 88% at 2 and 4 months, respectively, with a 1-year PFS of 66% (29).

In a prospective multicenter phase II study of sunitinib, 5 out of 19 patients (26.3%) achieved PD, while 8 (42.1%) achieved SD. The overall response rate was 26.3% (95% confidence interval [CI]: 6.3~45.7). With a median follow-up time of 20.3 months (1.8 ~ 50.7 months), the 2-year progression-free survival rate was 74.7% (30). The ORR of anlotinib (21.21%) was lower than that of sunitinib (26.3%), but the rate of PD was lower for anlotinib than for sunitinib, and the rate of SD was greater for anlotinib than for sunitinib. Compared with those of patients receiving sorafenib, in a prospective observational study involving 104 patients receiving sorafenib, the PFS rates at 1 year and 2 years were 86.6% (79.6 ~ 92.7%) and 73.7% (62.4 ~ 82.8%), respectively (13). The 1-year PFS of patients receiving anlotinib was 89.71%, and the 3-year PFS was 82.81%, which was greater than that of patients receiving sorafenib.

Nevertheless, notable toxicity and adverse reactions, including hand, foot, and skin reactions (89.4%), fatigue (79.8%), and alopecia (70.1%), were observed. Compared with sorafenib and sunitinib, pazopanib has a greater incidence of nausea, diarrhea, and hypertension (31). Reactive lymphadenopathy has been documented subsequent to treatment with pazopanib and imatinib (32). In our study, the incidence of hand-foot skin syndrome, hypertension, and diarrhea was greater in patients with desmoid fibromatosis than in patients with other soft tissue sarcomas treated with anlotinib. Rash and pharyngalgia were less common. Overall, most of the adverse reactions were Grade 1–2, and only 7.6% of the patients discontinued the drug because of drug toxicity. In contrast, drug-related toxicity leads to treatment discontinuation in 20% to 45% of patients receiving sorafenib or imatinib (33). These results suggest that patients tolerate anlotinib well, allowing long-term treatment to control tumor growth and achieve the goal of tumor stability.

In this retrospective study, although no patients achieved CR, only 4 patients achieved PD, the ORR was 21.21%, and the DCR reached 90.91%, indicating that anlotinib was not effective in the treatment of DF but had a strong ability to stabilize the tumor size. The safety of the 8 mg dose of anlotinib for the treatment of DF was initially explored in our previous review. The effects of different doses of DF were also investigated in this study. According to (RECIST) 1.1, there was no significant difference in efficacy among the three doses of anlotinib. The number of PRs decreased, and the number of PDs increased, which also reflected the effect of the dose on tumor size. However, from the perspective of drug side effects, 41.67% of patients with an initial dose of 12 mg needed dose reduction, and 13.6% of patients with an initial dose of 10 mg needed dose reduction. The dose administered to 12 mg patients was greater than that administered to 10 mg patients (P < 0.05). This result also suggested that, when selecting the drug dose, patients may initially consider commencing treatment with 10 mg. Despite its minimal impact on efficacy compared to a higher dose, it can decrease the frequency of patient dose adjustments and the occurrence of adverse reactions.

The question of whether targeted drugs before or after surgery reduce postoperative recurrence remains unresolved. In our retrospective study of 66 patients, among those treated with anlotinib, 20 underwent surgery, with 19 continuing medications after surgery and one not taking medication after surgery; these patients experienced recurrence. Additionally, we reviewed 12 DF patients who were not treated with anlotinib before surgery. Among these patients, 11 continued after surgery, and one who did not, experienced recurrence. The two patients who did not receive anlotinib after surgery experienced recurrence. Although the follow-up period was short and there was no cohort study evaluating the impact of postoperative anlotinib on recurrence, this result also suggested that postoperative anlotinib may reduce recurrence.

Currently, there is no definitive research on the optimal dosage of anlotinib, primarily due to the unclear treatment mechanism of anlotinib in advanced soft tissue sarcoma. The approval of anlotinib in China for use in soft tissue sarcomas was based on findings from phase II (ALTER0203) and phase III randomized clinical trials (APROMISS), which demonstrated improved median PFS compared to a placebo (34, 35). Anlotinib is typically recommended at a dosage of 12 mg for the treatment of advanced soft tissue sarcomas based on studies of the pharmacokinetics, systemic distribution, and half-life of anlotinib at different doses. In this retrospective study, we analyzed the efficacy and adverse reactions associated with different doses of anlotinib in desmoid fibromatosis patients (36, 37). No significant differences in ORR or PFS were observed based on the initial dose. Additionally, nearly half of the patients receiving 12 mg required dose adjustments. Therefore, we hypothesize that a lower initial dose may be appropriate, as it is better tolerated without compromising efficacy. However, DF itself is indolent and may be managed with AS. The median follow-up time in our study was only approximately one year. The impact of different doses on survival rates requires validation through prospective clinical studies with larger patient cohorts and longer follow-up periods. Furthermore, the relationships between different doses and their resulting blood concentrations, as well as the molecular mechanisms of action of anlotinib and its effects on desmoid fibromatosis development, warrant further experimental validation.

It is crucial to acknowledge that this investigation is a single-arm, single-center retrospective study, lacking a control group for comparison. A single institution also needs to consider the source of patients. Most of the patients in our institution come from southwest China, which does not represent all patients. This limitation complicates the assessment of the true efficacy of anlotinib compared to that of other treatments or a placebo. Additionally, not all patients can reliably adhere to regular reviews due to geographical constraints or the impact of COVID-19. The retrieval of patients’ clinical data is susceptible to recall bias, and considering that patients are not monitored as closely as they are in clinical trials, there is potential for toxicities, including rash and hypertension, that may not be fully documented. Currently, the molecular mechanism of anlotinib, which involves abnormal Wnt/β-catenin signaling, Cyclin D1 overexpression, estrogen receptor-β expression, etc. (38), is a significant area of research. However, gene mutation testing was not conducted in this study due to financial constraints.

## Conclusion

In conclusion, anlotinib is effective at managing recurrent and symptomatic patients with DF. This result also suggested that postoperative anlotinib may reduce recurrence. However, it’s toxicity profile of anlotinib indicates a greater risk of HFSR and hypertension. Therefore, given the elevated rate of dose titration associated with the initial dose of 12 mg, implementing dosage reductions may help balance efficacy with side effects.

## Data availability statement

The raw data supporting the conclusions of this article will be made available by the authors, without undue reservation.

## Ethics statement

The studies involving humans were approved by the Ethics Committee of West China Hospital, Sichuan University. The studies were conducted in accordance with the local legislation and institutional requirements. Written informed consent for participation in this study was provided by the participants’ legal guardians/next of kin. Written informed consent was obtained from the individual(s), and minor(s)’ legal guardian/next of kin, for the publication of any potentially identifiable images or data included in this article.

## Author contributions

MX: Formal Analysis, Methodology, Writing – original draft. QH: Data curation, Investigation, Writing – original draft. TG: Writing – review & editing. YW: Writing – review & editing. ZL: Writing – review & editing. ML: Writing – review & editing. YL: Writing – review & editing. LM: Writing – review & editing. YZ: Writing – review & editing. CT: Writing – review & editing.
