# Supplementary figures and images for: Efficacy and safety of anlotinib in patients with desmoid fibromatosis: a retrospective analysis
Source: Front Oncol. 2024 May 14;14:1399574. doi: 10.3389/fonc.2024.1399574 (PMC11130419; doi:10.3389/fonc.2024.1399574)

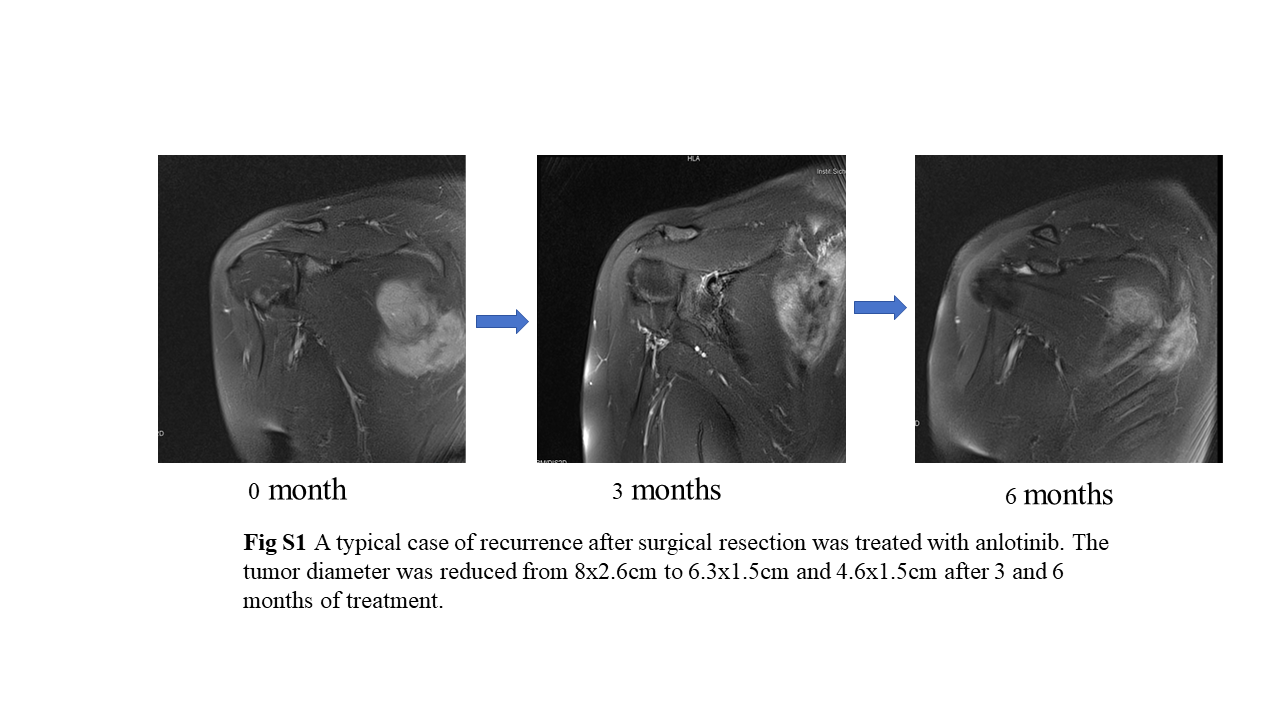

Supplement: Supplementary file 1 [file Image_1.tif]

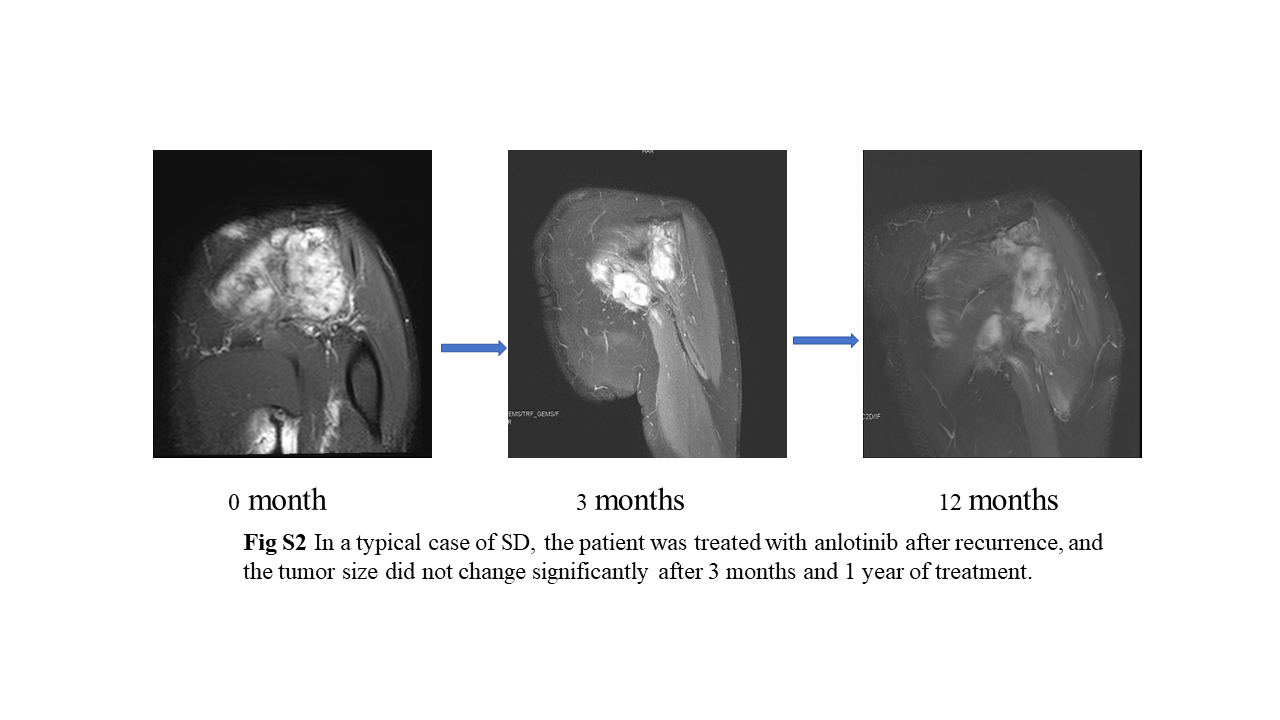

Supplement: Supplementary file 2 [file Image_2.tif]

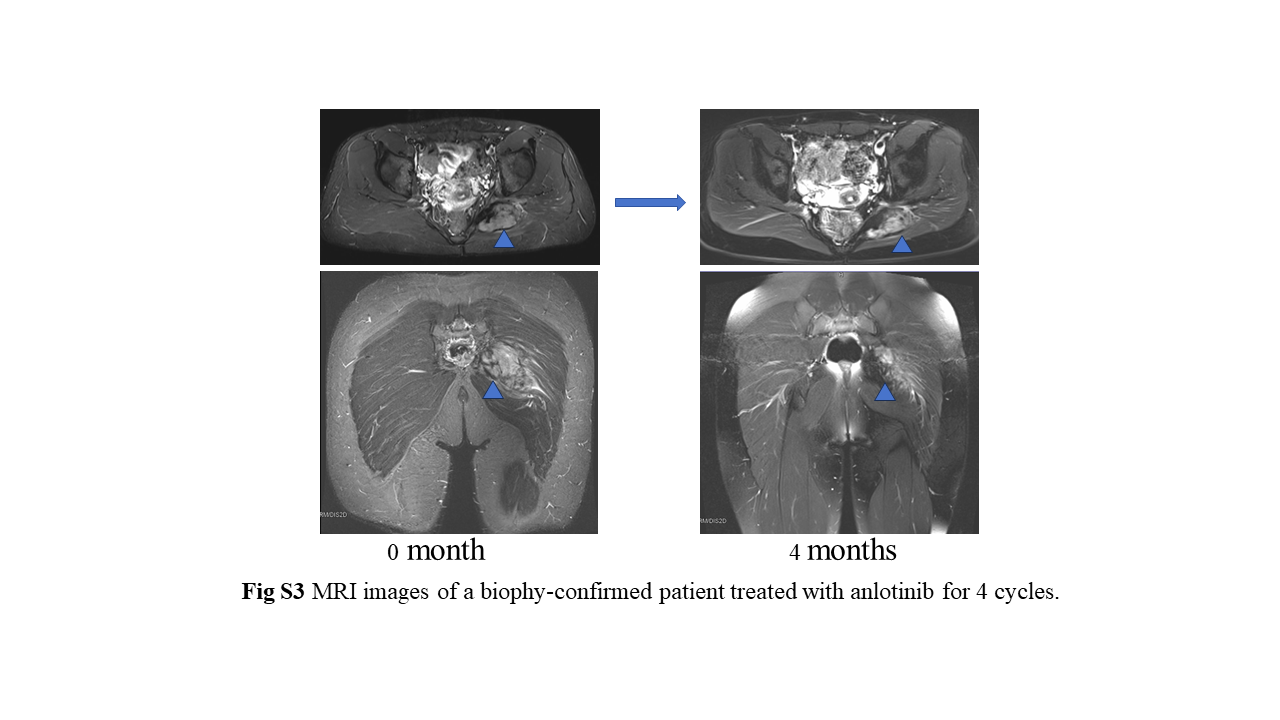

Supplement: Supplementary file 3 [file Image_3.tif]
